# Patient Navigation Plus Tailored Digital Video Disc Increases Colorectal Cancer Screening Among Low-Income and Minority Patients Who Did Not Attend a Scheduled Screening Colonoscopy: A Randomized Trial

**DOI:** 10.1093/abm/kaae013

**Published:** 2024-03-12

**Authors:** Susan M Rawl, Susan M Perkins, Yan Tong, Mira L Katz, Lisa Carter-Bawa, Thomas F Imperiale, Peter H Schwartz, Hala Fatima, Connie Krier, Kevin Tharp, Rivienne Shedd-Steele, Mark Magnarella, Caeli Malloy, Laura Haunert, Netsanet Gebregziabher, Electra D Paskett, Victoria Champion

**Affiliations:** Center for Research and Scholarship, School of Nursing, Indiana University at Indianapolis, Indianapolis, IN, USA; Cancer Prevention and Control Program, Indiana University Melvin and Bren Simon Comprehensive Cancer Center, Indianapolis, IN, USA; Cancer Prevention and Control Program, Indiana University Melvin and Bren Simon Comprehensive Cancer Center, Indianapolis, IN, USA; Department of Biostatistics and Health Data Science, Indiana University School of Medicine, Indianapolis, IN, USA; Cancer Prevention and Control Program, Indiana University Melvin and Bren Simon Comprehensive Cancer Center, Indianapolis, IN, USA; Department of Biostatistics and Health Data Science, Indiana University School of Medicine, Indianapolis, IN, USA; Department of Health Behavior and Health Promotion, College of Public Heath, The Ohio State University (OSU), Columbus, OH, USA; Cancer Control Program, Comprehensive Cancer Center, The Ohio State University (OSU), Columbus, OH, USA; Community Outreach and Engagement, Center for Discovery & Innovation, Cancer Prevention Precision Control Institute, Hackensack Meridian Health, Nutley, NJ, USA; Department of Medicine, Indiana University School of Medicine, Indianapolis, IN, USA; Department of Medicine, Indiana University School of Medicine, Indianapolis, IN, USA; Department of Medicine, Indiana University School of Medicine, Indianapolis, IN, USA; Center for Research and Scholarship, School of Nursing, Indiana University at Indianapolis, Indianapolis, IN, USA; Indiana University Center for Survey Research, Bloomington, IN, USA; Cancer Prevention and Control Program, Indiana University Melvin and Bren Simon Comprehensive Cancer Center, Indianapolis, IN, USA; Eo Studios, Athens, GA, USA; Center for Research and Scholarship, School of Nursing, Indiana University at Indianapolis, Indianapolis, IN, USA; School of Health and Human Sciences, Physician Assistant Program, Indiana University at Indianapolis, Indianapolis, IN, USA; Department of Biostatistics and Health Data Science, Indiana University School of Medicine, Indianapolis, IN, USA; Cancer Control Program, Comprehensive Cancer Center, The Ohio State University (OSU), Columbus, OH, USA; Division of Cancer Prevention and Control, Department of Medicine, College of Medicine, The Ohio State University, Columbus, OH, USA; Center for Research and Scholarship, School of Nursing, Indiana University at Indianapolis, Indianapolis, IN, USA; Cancer Prevention and Control Program, Indiana University Melvin and Bren Simon Comprehensive Cancer Center, Indianapolis, IN, USA

**Keywords:** Patient navigation, Tailored intervention, Colorectal cancer screening, Minorities, Low income

## Abstract

**Background:**

Up to 50% of people scheduled for screening colonoscopy do not complete this test and no studies have focused on minority and low-income populations. Interventions are needed to improve colorectal cancer (CRC) screening knowledge, reduce barriers, and provide alternative screening options. Patient navigation (PN) and tailored interventions increase CRC screening uptake, however there is limited information comparing their effectiveness or the effect of combining them.

**Purpose:**

Compare the effectiveness of two interventions to increase CRC screening among minority and low-income individuals who did not attend their screening colonoscopy appointment—a mailed tailored digital video disc (DVD) alone versus the mailed DVD plus telephone-based PN compared to usual care.

**Methods:**

Patients (*n* = 371) aged 45–75 years at average risk for CRC who did not attend a screening colonoscopy appointment were enrolled and were randomized to: (i) a mailed tailored DVD; (ii) the mailed DVD plus phone-based PN; or (iii) usual care. CRC screening outcomes were from electronic medical records at 12 months. Multivariable logistic regression analyses were used to study intervention effects.

**Results:**

Participants randomized to tailored DVD plus PN were four times more likely to complete CRC screening compared to usual care and almost two and a half times more likely than those who were sent the DVD alone.

**Conclusions:**

Combining telephone-based PN with a mailed, tailored DVD increased CRC screening among low-income and minority patients who did not attend their screening colonoscopy appointments and has potential for wide dissemination.

## Teaser

Combining telephone-based patient navigation with a mailed, tailored digital video disc increased CRC screening by four-fold among low income and minority patients who did not attend their screening colonoscopy appointments.

## Introduction

The United States Preventive Services Task Force (USPSTF) recommends screening for colorectal cancer (CRC) beginning at age 45 [1]. There are several options for CRC screening for average risk individuals including: annual stool testing with fecal occult blood tests or fecal immunochemical tests (FIT), stool DNA-FIT test every 1–3 years, sigmoidoscopy every 5 years, or colonoscopy every 10 years [1]. For people with a strong family history of CRC or polyps, colonoscopy is the most appropriate screening test. Therefore, the recommended CRC screening test should be based on assessment of an individual’s risk factors [2, 3]. Unfortunately, the prevalence of CRC screening, while increasing, remains below the national goal of 80% [4] particularly for low-income and minority populations [5].

Overall, from 14% to 67% of people referred for colonoscopy cancel or do not attend their scheduled appointments [6–11]. Low-income and minority patients served by safety net healthcare systems often do not complete colonoscopies for which they have been referred and scheduled [6, 9, 12]. Even for people who present for colonoscopy, inadequate bowel preparation is a major impediment to effective CRC screening with colonoscopy, with 25%–30% of patients presenting for colonoscopy not adequately cleansing, or emptying, their bowel [9, 13–15]. Suboptimal cleansing of the bowel leaves residual fecal matter that obscures polyps, leads to missed diagnoses, extends the time needed for the examination, and results in incomplete or aborted colonoscopies [9, 14–18]. Poor bowel preparation has been estimated to increase the cost of colonoscopy by 22% in public hospitals due to patients needing to repeat a colonoscopy or have another test earlier than recommended by current practice guidelines [17]. Finding effective approaches to help patients who are willing to have a colonoscopy prepare for that test can reduce costs for patients and health systems, an especially important goal for those with limited resources.

While several CRC screening test options exist for people at average risk, providers often recommend colonoscopy and do not offer alternative screening strategies [19, 20]. A growing body of evidence demonstrates that offering patients different test options substantially increases adherence to screening recommendations [21]. While colonoscopy is the most appropriate test for individuals at increased risk for CRC, those at average risk—the majority of the population—can be screened with tests that are noninvasive and less resource intensive (i.e., time and cost). If those at average risk were given a choice of tests by providers, adherence to screening would increase [19, 20]. The common practice of only recommending colonoscopy may create a barrier and reduce CRC screening adherence, especially among racial/ethnic minorities [19]. To further reduce CRC incidence and mortality, provider referral and timely completion of CRC screening tests that include, but are not limited to, colonoscopy are imperative.

Tailored interventions, defined as “any combination of information and behavior change strategies intended to reach one specific person, based on characteristics that are unique to that person, related to the outcome of interest, and derived from individual assessment” are effective approaches to increase CRC screening [22–28]. Kreuter et al. first categorized health messages on a continuum from generic to interpersonal based on the level of assessment required. Tailored health communications can be delivered using a variety of media but computer technology is required for the tailoring process [29]. Computers are used to generate tailored messages based on user responses to queries within programs. While humans (e.g., healthcare providers) can deliver highly individualized messages, these messages are classified as “interpersonal,” rather than tailored, because they are based on interpersonal interactions and require a higher level of assessment of the recipient. Tailored interventions have influenced health behavior change in relation to smoking cessation, dietary change, physical activity, mammography, and CRC screening [30–35] and are more effective than nontailored interventions that do not take into account individual characteristics of intervention recipients [36, 37]. Studies have shown that tailored interventions eliminate unnecessary information that does not apply to the patient and are more: (i) personally relevant to the user; (ii) likely to be paid attention to; (iii) likely to lead to thoughtful consideration of behavior change; and (iv) useful than nontailored information in helping people enact behavior change [38].

High-quality evidence supports patient navigation (PN) as an effective approach to increasing uptake of CRC screening [10, 39–46]. PN in the context of cancer has been defined as a barrier reduction-focused intervention that: (i) is provided to individual patients for a defined episode of cancer-related care; (ii) has a definite endpoint when provision of services is complete; (iii) targets a defined set of services to complete an episode of cancer-related care; (iv) focuses on reducing patient-level barriers to accessing care; and (v) reduces delays in accessing care with an emphasis on timeliness and reduction in the number of patients lost to follow-up [47]. The effectiveness of PN for increasing cancer screening rates has been well established and numerous studies have supported the effectiveness of PN interventions to increase CRC screening [39, 40, 48–56]. A comprehensive review concluded that navigation was an effective approach to increasing cancer screening based on four studies focused on CRC screening, three on breast cancer screening and one on cervical cancer screening [57].

Both computer-tailored small media (digital video disc, DVD) and PN interventions have been shown to increase uptake of CRC screening and improve bowel preparation, but only one other study has evaluated the additive effect of combining these two approaches for CRC screening [58]. People differ on the types and levels of intensity of interventions needed to move them to complete CRC screening [59]. In this study, we compared the effectiveness of two theory-guided, evidence-based interventions—a tailored DVD alone compared to a tailored DVD plus telephone-based PN compared to usual care—to increase CRC screening among low-income and minority patients who did not keep their appointment for a screening colonoscopy.

## Purpose/Objectives

The purpose of this randomized controlled trial was to compare the effectiveness of two theory-based interventions to increase CRC screening completion among low-income and minority patients who were referred and scheduled for a screening colonoscopy but did not attend their appointment. The trial was conducted between August 2017 and October 2020 and recruited patients being seen in a single safety net health system. The aim of the study was to:

Compare the effectiveness of two interventions designed to promote CRC screening among people at average risk for CRC—a tailored DVD versus the tailored DVD *plus* telephone-based PN—to each other and to usual care.

*Hypothesis 1*: Participants who receive the tailored DVD *plus* telephone-based PN intervention will have higher rates of CRC screening with FIT, colonoscopy, or either screening test compared to those who receive only the tailored DVD.

*Hypothesis 2*: Participants who receive either intervention will have higher rates of CRC screening with FIT, colonoscopy, or either screening test than those who receive usual care.

*Hypothesis 3*: Participants who receive either intervention who complete colonoscopy will have: (i) better quality of bowel preparation; (ii) less anxiety about the procedure; and (iii) greater satisfaction with the colonoscopy experience than those who receive usual care.

## Design and Methods

**Fig. 1.**
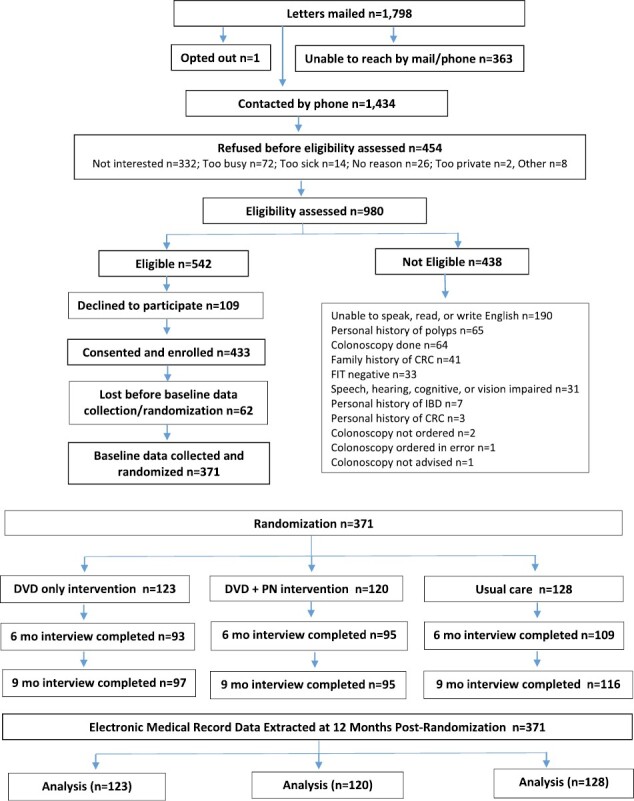
CONSORT diagram.

Potentially eligible individuals who were scheduled for screening colonoscopy in a safety net health system, but did not attend their appointment, were sent a letter signed by the endoscopy medical director inviting them to participate in the study. For those who did not call to decline participation, trained research staff called potential participants to assess interest, explain the study, answer questions, and determine eligibility. From those who were eligible and willing to participate, verbal consent was obtained. After baseline data were collected via structured telephone interviews, participants were randomized to receive either: (i) a mailed interactive DVD; (ii) the mailed DVD *plus* a telephone-based PN intervention; or (iii) usual care (Fig. 1). All study procedures were approved by the Indiana University Institutional Review Board and compliant with both clinic and Health Insurance Portability and Accountability Act (HIPAA) requirements.

## Interventions

### DVD Intervention

The interactive tailored DVD intervention was a refinement of a tablet-based computerized tailored intervention that we had previously developed to promote CRC screening in African Americans [26]. We refined it in collaboration with a multimedia design company, the study’s Community Advisory Board, and our research team which included gastroenterologists and experts in health communication. While the prior version of the intervention was built on a tablet-based platform (for delivery in primary care clinics), the program was redesigned to be delivered as an interactive DVD that could be mailed to participants’ homes for this study. This approach was based on a preliminary study showing that internet access was extremely limited among members of the target audience in this community while DVD players were commonly available. In addition, prior to beginning recruitment for the study, we pretested the tailored DVD intervention for usability and satisfaction with five individuals who were representative of our target population.

The goal of the tailored DVD intervention was to help participants make an informed decision about the importance of CRC screening, preparing for, and completing a screening test. The narrative theme of the interactive tailored DVD was a house call by a doctor to talk about what to do to stay healthy, namely getting screened to prevent colon cancer. A professional actor played the role of the doctor who narrated the program; minimal reading was required to engage with the program. On average, the DVD required 20 min to view. Individual sections (or the entire DVD) could be replayed by using a chapter menu at the end of the program. The first section focused on the gastrointestinal system, how CRC develops, CRC risk factors, and benefits of testing. Benefits were emphasized by two testimonials by CRC survivors who, like most people, did not think they were at risk for CRC. These were followed by brief descriptions of the two most common CRC screening tests: colonoscopy and stool-based test.

The next section focused specifically on colonoscopy. Video demonstrating how to complete the bowel preparation was tailored based upon the type of bowel preparation ordered for the participant (GoLYTELY/NuLYTELY or Miralax). Barriers to completing colonoscopy were then assessed with participants indicating “Yes” or “No” using their DVD remote control. For each barrier endorsed, a tailored testimonial designed to address the specific barrier was delivered by a patient or healthcare provider. The colonoscopy section concluded by asking participants if they intended to get a colonoscopy within the next 6 months or if they would like to hear more about the stool-based test as another option.

Participants who elected to learn about stool-based testing viewed the next section which was an in-depth description of FIT—the stool-based test used in this health system. Contents of the FIT kit were shown along with a detailed demonstration of how to collect a stool sample and complete the test. The DVD program then assessed common barriers to stool-based testing with participants indicating agreement with each barrier that was relevant to them using their remote control. For each barrier endorsed, a tailored testimonial to overcome the barrier was delivered by a patient or healthcare provider. A summary of the advantages and disadvantages of the FIT test and colonoscopy was presented, and participants were asked to select which test—colonoscopy or stool-based test—they were most likely to do in the next 6 months.

The final section of the program concluded with the doctor congratulating participants on completing the program and encouraging them to get tested soon to stay healthy. The program concluded with instructions on how to schedule a colonoscopy or request an FIT kit. We conducted a comprehensive process evaluation and reported the development of the DVD, viewership, engagement, participant perceptions of relevance and satisfaction with the DVD [60]. Engagement was relatively high with 84% of participants having watched the DVD and 99% reporting that the DVD was very or somewhat helpful.

### PN Intervention

The PN intervention consisted of individualized counseling delivered by telephone by a trained registered nurse. Training addressed: (i) the principles of PN; (ii) patient-identified barriers to CRC screening; (iii) health literacy as a barrier; (iv) roles and responsibilities of the navigator; (v) effective communication using open-ended questions, affirmations, reflective listening, and summaries; (vi) patient empowerment and self-efficacy to complete CRC screening; and (vii) teach back methods to check for understanding. The navigators watched the tailored DVD intervention in order to learn what CRC information participants would receive in advance of navigation. Training then focused on how to conduct the PN calls designed to reduce barriers, advocate for testing, prepare for a colonoscopy or complete an FIT, and to provide encouragement and support. This was followed by case studies so that the nurses could practice delivering the navigation intervention.

Navigators followed a protocol embedded in a dedicated REDCap database to guide and document details about each PN intervention session. This database provided a standardized framework for the phone conversation with the flexibility to personalize the content based upon participants’ expressed needs and concerns. Phone calls and mailings to participants, as well as contacts made on their behalf with the endoscopy scheduler, clinical nurse manager, or endoscopy nurse, were documented in this database. With the permission of participants, PN sessions were audio recorded and a subsample evaluated for intervention fidelity. If participants preferred that conversations not be recorded, the navigator documented the reason, if volunteered, and proceeded with the intervention.

Navigators placed an initial call to participants randomized to the DVD + PN intervention arm 2 weeks after mailing the DVD. The initial call began with the navigator confirming that participants had not already rescheduled and completed a colonoscopy. Next, the navigator assessed receipt of the mailed DVD and whether or not participants had viewed it. If participants had not yet viewed the DVD, the navigator reminded them to do so, confirmed that participants had a working DVD player, and rescheduled the call. When needed, the navigator mailed a second DVD. The navigator also arranged for DVD players to be loaned to 23 participants who reported not having one or experiencing technical difficulties with their own equipment. If participants had viewed the DVD, the navigator asked if they had any questions and answered them. Navigators then counseled participants to increase their knowledge of CRC screening, risk factors, and the advantages and disadvantages of each screening test, then focused on assessing and reducing cognitive and logistical barriers to CRC screening. The protocol provided guidance to navigators to address common CRC barriers including: (i) no bowel problems; (ii) no family history; (iii) embarrassment; (iv) fear of finding cancer; (v) cost and insurance issues; (vi) time constraints; (vii) bowel preparation for colonoscopy; (viii) transportation; (ix) pain or discomfort associated with a colonoscopy; and (x) collecting a stool sample is messy. When participants denied having any barriers, the navigator gently probed by asking about common barriers such as having the time to do the test and cost. After addressing barriers, the navigators asked participants if they would like to reschedule the colonoscopy or preferred a stool test. For participants who were ready to reschedule, the navigator notified the Endoscopy Department to reschedule a colonoscopy. For participants who preferred an FIT, the navigators mailed an FIT kit to their home. If a participant was undecided about which screening test to have, the navigators arranged to call the participant back at a later date.

The navigators called participants who rescheduled their colonoscopy 1–3 days prior to the appointment to confirm that participants had received written instructions on how to prepare for the procedure from the Endoscopy Department. The navigator then reviewed the colonoscopy procedure, explained what participants would experience, and walked participants through the bowel preparation. This included what was allowed on a clear liquid diet, how to mix the GoLYTELY/NuLYTELY or Miralax solutions, tips to make drinking the solution more tolerable, and timeline for doing the preparation. The navigator also addressed how to manage or adjust current medications patients were taking (e.g., insulin and anticoagulants) and confirmed that transportation was arranged. For participants who were mailed an FIT kit, the navigator called 1–2 weeks after mailing to confirm receipt, review the process for collecting a stool sample, answer questions, encourage completion, and remind participants to return the kit to their primary care clinic.

### Usual Care

All participants in the study received usual care. Both intervention groups received their assigned intervention in addition to usual care. In the Endoscopy Department, patients had their colonoscopy appointment automatically set after their primary care provider referred them through the electronic medical record (EMR). Then, patients were mailed a packet of information that included a letter showing their appointment time, instructions for preparing, and a number to call with questions. If patients were unable to attend their appointment, they needed to call the Endoscopy Department to change or cancel. A nurse from the Endoscopy Department telephoned patients a day or two before their appointment to remind them and answer questions. Most often, the nurse left a private voicemail message as they were frequently unable to reach participants by phone. Patients who canceled or did not attend their colonoscopy appointment were contacted by the endoscopy scheduler to reschedule their appointment. If a patient was reached and declined to reschedule the colonoscopy, then no further contact was made.

## Recruitment

Participants were eligible if they had: (i) been referred and scheduled for a screening colonoscopy that was not completed in the past week; (ii) were 45–75 years old; (iii) able to speak, read, and write English. Patients who had an FIT with positive results in the past 12 months were eligible since a positive FIT must be followed by colonoscopy to complete the screening process. Patients were excluded if they had: (i) a personal history of CRC or adenomatous polyps; (ii) a personal history of conditions that increased CRC risk (ulcerative colitis, Crohn’s disease, or known hereditary syndromes); (iii) a family history which increased CRC risk; or (iv) any speech, hearing, cognitive, or vision impairments.

After potentially eligible patients were approved for contact by the endoscopy nurse manager, trained research staff mailed 1,798 introductory letters signed by the endoscopy medical director along with a brochure that explained the study. The letter informed patients they would receive a phone call about the study within the next week unless they called a toll-free number to opt-out. Approximately 1 week after letters were mailed, trained recruiters reached 1,434 (79.8%) patients by phone who did not opt-out to explain study requirements, potential risks, compensation, and answer questions. Of 1,434 who were reached, eligibility was assessed in 980 (68.3%) patients as 454 (31.7%) refused participation before eligibility was determined. Among these 454, reasons for refusal of eligibility assessment included not being interested (*n* = 332, 63.1%), too busy (*n* = 72, 13.7%), too sick (*n* = 14, 2.7%), and other reasons (*n* = 36, 6.8%). Among the 980 whose eligibility was assessed, 542 (55.3%) met eligibility criteria. Among 542 who were eligible, recruiters obtained informed consent and permission to access medical records from 433 (79.9%) and scheduled a convenient time for the baseline interview. Of those 433 who consented, 371 (85.7%) were able to be reached by phone to complete the baseline interview and be randomized (see Fig. 1). Recruitment began on July 26, 2017 and end-of-study data were collected for the final participant by October 21, 2020. The final participant was randomized in October 2019 and received the intervention within a few weeks after randomization, several months before COVID began to impact colonoscopy appointments in March 2020. Since we extracted screening outcomes from the EMR at 12 months post-randomization, screening outcomes for the final participant were extracted in October 2020.

## Data Collection

Baseline self-reported data were collected within 2 weeks of enrollment by trained interviewers employed by the Indiana University Center for Survey Research using computer-assisted telephone interviews. Interviews averaged 46 min in duration. Randomization to an intervention arm or usual care occurred by computer at the completion of the baseline interview. Follow-up interviews were conducted at 6 months with participants who completed colonoscopy to assess colonoscopy-related procedure anxiety and satisfaction with the colonoscopy experience. Our primary outcome was participant completion of any CRC screening test defined as either colonoscopy or FIT within 12 months after randomization as documented in the EMR. The quality of bowel preparation (cleanliness of the bowel) score also was extracted from the EMR as evaluated by the endoscopist during the procedure as an exploratory aim.

## Measures

### Demographics and Clinical Variables

Demographic variables included sex, race, ethnicity, marital/partnered status, health insurance, education, employment, annual household income, perceived income adequacy, age, and body mass index. *Health status* was assessed using the 10-item Patient-Reported Outcome Measures Information System (PROMIS) Global Health short form [61]. The scale includes physical health and mental health subscales that demonstrated Cronbach alpha reliability coefficients of 0.76 and 0.78, respectively. *Health literacy* was measured using the 3-item Brief Health Literacy Screen [62]. This scale had a Cronbach alpha of 0.69 in this study. *Comorbidities* were assessed using the 12-item Self-Reported Comorbidity Questionnaire developed by Sangha et al. [63] which obtained a Cronbach’s alpha of 0.76.

*Colonoscopy-related procedural anxiety* was measured using the 6-item short form of the State Anxiety Scale of the State-Trait Anxiety Inventory [64]. This scale measured how an individual feels when thinking about having a colonoscopy using response options where 1 = not at all, 2 = somewhat, 3 = moderately so, and 4 = very much so. Cronbach’s alpha in this study was 0.62. *Satisfaction with the colonoscopy experience* was measured by self-report using a single item developed by the research team. Participants rated how satisfied they were on a 4-point scale where 1 = not at all satisfied, 2 = a little satisfied, 3 = mostly satisfied, and 4 = completely satisfied.

*Quality of bowel preparation* was extracted from the EMR. Endoscopists used a modified Aronchick bowel preparation rating scale where they rated the quality of the bowel preparation as excellent, good, fair, adequate, poor, or inadequate [65]. Ratings of excellent, good, fair, or adequate were coded as “adequate” while ratings of poor or inadequate were coded as “inadequate.” Endoscopists also rated the quality of bowel preparation using the well-validated Boston Bowel Preparation Scale (BBPS) [66]. The BBPS is a 10-point scale, scored from 0 to 9 where 0 = unprepared colon with mucosa not seen and 9 = entirely clean colon.

## Data Analysis

Descriptive analyses compared baseline demographic information across groups using means and standard deviations for continuous variables and frequency distributions for categorical variables. This included the calculation of standardized differences as described in Yang and Dalton [67]. For dichotomous variables [68], Austin recommends a cutoff standardized difference of >0.10 for declaring imbalance. For continuous variables [69], Austin recommends values of 1.96*sqrt(2/*n*) where *n* is the common group sample size. Our average sample size per study arm was *n* = 126, so the cutoff would be >0.25. There are no guidelines for nominal or ordinal variables, so we used 0.20 for the cutoff. With regard to missing data, within-subject mean imputation was used for items that were missing for scale scores as long as two-thirds of the items on the scale or subscale were answered. The exceptions were the global mental health, global physical health, and comorbidity scales, where no imputation was done per instruments’ instructions. Variables that had standardized differences larger than the suggested cutoffs, were either controlled for a priori (race, combined with Hispanic ethnicity) or were included in multivariable models when associated with the outcome univariately (years of education, have health insurance, global mental health score, and comorbidity scale score).

Primary analysis of the EMR-documented screening outcomes employed an intent-to-treat analysis (analyze as randomized); all participants were included regardless of adherence with interventions or loss to follow-up. Characteristics of participants who dropped out by 9 months were compared with those who did not using analysis of variance (ANOVA), chi-square tests, or exact or nonparametric equivalents. No significant differences in any variables at baseline between those who dropped out versus those who did not were observed. There were no missing data for our EMR-based screening outcome measures (screening test done or not done).

We tested the primary aim and related hypotheses to compare intervention effects among the three randomized arms using the primary outcome of completion of CRC screening by *any test* (FIT or colonoscopy) at 12 months post-baseline as documented in the EMR. We also examined completion of FIT or colonoscopy independently. As an initial assessment of CRC screening completion at 12 months, we used a chi-square test for a 3 × 2 (arm by screening completion) contingency table to test whether completion rates differed across arms. If the *p* value for the overall test was significant at the .05 level, pairwise 2 × 2 tests were conducted at the 0.05/3 = 0.017 level. Multivariable logistic regression models of CRC screening test completion at 12 months (yes/no) were also conducted. Independent variables were arm assignment, age category, sex, and race/Hispanic ethnicity (all used in the stratified randomization) and continuous and categorical baseline individual characteristics if they were related to the outcome in univariate analyses (using *p* < .20 as the cutoff). Arm assignment was coded using dummy variables with usual care as the reference category. Quality of bowel preparation was analyzed for those participants who completed colonoscopy with a χ^2^ test for the modified Aronchick score (adequate vs. not adequate) and ANOVA for the continuous outcome BBPS score. Colonoscopy-related procedure anxiety and satisfaction with the procedure were compared using ANOVA.

## Results

### Sample Characteristics

Demographic characteristics of the total sample and by group assignment are displayed in Table 1. Participants had a mean age of 57.8 (standard deviation = 6.0) years, with 86.0% being less than 65 years of age. They were primarily female (*n* = 225, 60.6%), Black or African American (*n* = 244, 65.8%), non-Hispanic (*n* = 358, 96.5%), and not married or partnered (*n* = 256, 69.0%). Most participants had some form of health insurance (*n* = 349, 94.1%) and had completed high school or more years of education (*n* = 285, 76.8%). The majority reported they were not currently employed (*n* = 236, 63.6%), had an annual household income of <15K (*n* = 154, 41.5%) and almost half (*n* = 172, 46.3%) said they had difficulty paying their bills or had to cut back on things to pay bills. The mean (standard deviation) for body mass index (BMI) was 31.10 (8.6), and the mean Global Physical Health scale score was 12.96 (3.3), Global Mental Health scale score was 12.82 (3.3), Health Literacy scale score was 12.03 (2.9), and Comorbidity scale score was 7.84 (4.8).

**Table 1:** Baseline Demographic Characteristics of All Participants and by Study Arm

Characteristics	Study arm			
	Overall *n* = 371	DVD only *n* = 123	DVD + PN *n* = 120	Usual care *n* = 128
	*n* (%)	*n* (%)	*n* (%)	*n* (%)
Sex				
Male	146 (39.4%)	48 (39.0%)	47 (39.2%)	51 (39.8%)
Female	225 (60.6%)	75 (61.0%)	73 (60.8%)	77 (60.2%)
Race				
Black or African American	244 (65.8%)	80 (65.0%)	77 (64.2%)	87 (68.0%)
White	105 (28.3%)	35 (28.5%)	33 (27.5%)	37 (28.9%)
Other	17 (4.6%)	6 (4.9%)	8 (6.7%)	3 (2.3%)
Hispanic ethnicity				
No	358 (96.5%)	119 (96.7%)	116 (96.7%)	123 (96.1%)
Yes	10 (2.7%)	3 (2.4%)	3 (2.5%)	4 (3.1%)
Race/ethnicity^a^				
Non-Hispanic White	104 (28.0%)	35 (28.5%)	33 (27.5%)	36 (28.1%)
Non-Hispanic Non-White	252 (67.9%)	83 (67.5%)	82 (68.3%)	87 (68.0%)
Other	15 (4.0%)	5 (4.1%)	5 (4.2%)	5 (3.9%)
Married/partnered				
No	256 (69.0%)	85 (69.1%)	83 (69.2%)	88 (68.8%)
Yes	114 (30.7%)	38 (30.9%)	36 (30.0%)	40 (31.3%)
Have health insurance				
No	21 (5.7%)	9 (7.3%)	6 (5.0%)	6 (4.7%)
Yes	349 (94.1%)	114 (92.7%)	114 (95.0%)	121 (94.5%)
Education				
11th grade or less	85 (22.9%)	28 (22.8%)	22 (18.3%)	35 (27.3%)
12th grade/HS diploma/GED	129 (34.8%)	42 (34.1%)	41 (34.2%)	46 (35.9%)
Vocational school/some college	118 (31.8%)	39 (31.7%)	45 (37.5%)	34 (26.6%)
College grad/graduate degree	38 (10.2%)	14 (11.4%)	12 (10.0%)	12 (9.4%)
Employment status				
Not employed	236 (63.6%)	83 (67.5%)	66 (55.0%)	87 (68.0%)
Employed full time	81 (21.8%)	22 (17.9%)	35 (29.2%)	24 (18.8%)
Employed part time	53 (14.3%)	18 (14.6%)	19 (15.8%)	16 (12.5%)
Household income				
Less than $15,000	154 (41.5%)	57 (46.3%)	45 (37.5%)	52 (40.6%)
$15,001 to $30,000	130 (35.0%)	46 (37.4%)	40 (33.3%)	44 (34.4%)
$30,001 to $50,000	48 (12.9%)	13 (10.6%)	20 (16.7%)	15 (11.7%)
$50,001 to >$100,000	26 (7.0%)	5 (4.1%)	9 (7.5%)	12 (9.4%)
Adequacy of income				
After paying the bills, you have enough money for special things that you want	53 (14.3%)	17 (13.8%)	16 (13.3%)	20 (15.6%)
You have enough money to pay bills, but little spare money to buy extra or special things	129 (34.8%)	44 (35.8%)	41 (34.2%)	44 (34.4%)
You have money to pay bills, but only because you cut back on things	65 (17.5%)	22 (17.9%)	22 (18.3%)	21 (16.4%)
You have difficulty paying the bills, no matter what you do	107 (28.8%)	36 (29.3%)	34 (28.3%)	37 (28.9%)

	Mean (*SD*)	Mean (*SD*)	Mean (*SD*)	Mean (*SD*)
Age	57.78 (5.96)	58.20 (6.07)	57.29 (5.63)	57.83 (6.17)
Body mass index^b^	31.10 (8.59)	31.57 (8.89)	30.18 (7.86)	31.50 (8.93)
Global Physical Health^c^^,^^d^ scale score	12.96 (3.31)	12.64 (3.24)	13.37 (3.29)	12.89 (3.38)
Global Mental Health^e^^,^^f^ scale score	12.82 (3.32)	12.86 (3.19)	12.73 (3.50)	12.88 (3.31)
Health Literacy^g^^,^^h^ scale score	12.03 (2.87)	12.20 (2.74)	11.97 (2.64)	11.91 (3.18)
Comorbidity^i^ scale score	7.84 (4.80)	8.24 (4.86)	7.73 (4.83)	7.56 (4.72)

*DVD* digital video disc; *GED* General Education Development; *HS* high school; *PN* patient navigation; *SD* standard deviation.

^a^Non-Hispanic Non-White included 242 Non-Hispanic Black and 10 Non-Hispanic other race participants, Other included 10 Hispanic participants (2 Hispanic Black, 1 Hispanic White, 7 Hispanic other race) and 5 participants who declared do not know or refused to answer their race or Hispanic ethnicity.

^b^ *n* = 3 missing for DVD + PN.

^c^Global Physical Health scale score has 4 items (from PROMIS Global Short Form questionnaire) with range 4–20, where higher scores indicate better physical health.

^d^ *n* = 1 missing for DVD + PN; *n* = 3 missing for usual care.

^e^Global Mental Health scale score has 4 items (from PROMIS Global Short Form questionnaire) with range 4–20, where higher scores indicate better mental health.

^f^ *n* = 2 missing for DVD + PN.

^g^Health Literacy scale score has 3 items and possible scores ranging from 1 to 15, where higher scores indicate higher literacy levels.

^h^ *n* = 1 missing for DVD + PN.

^i^Comorbidity scale score has 36 items (representing 12 comorbidities) and possible scores range from 0 to 36, where higher scores indicate greater comorbidity.

### CRC Screening Test Documented in the EMR

With regard to the primary outcome of CRC screening with *any test* (colonoscopy or FIT) as documented in the EMR, our hypothesis that screening rates would be higher in the intervention arms than those who received usual care was only partially supported. As shown in Table 2, participants who received the DVD + PN intervention had significantly higher rates of any CRC screening (49.2%) than those who received the DVD alone (30.1%) or Usual Care (21.1%; *p* < .001). Despite being 9% higher, the screening rate with any CRC screening test for those in the DVD only arm was not significantly different from usual care (30.1% vs. 21.1%; *p* = .102). In addition to the primary outcome of any CRC screening test, colonoscopy, and FIT screening rates as documented in the EMR were examined independently. The DVD + PN arm had higher rates of colonoscopy screening (38.3%) than both DVD only (17.9%) and usual care (14.8%; *p* < .001). DVD only was not different from usual care (*p* = .515). For FIT screening, the differences by treatment group were not significant (*p* = .248).

**Table 2:** EMR-Documented CRC Screening (Colonoscopy, FIT, or Both) by Total Participants and by Study Arm

Screening	Study arm				Overall *X*^2^ *p* value	DVD + PN vs. DVD only Difference (98.3% CI) *X*^2^ *p* value	DVD only vs. usual care Difference (98.3% CI) *X*^2^ *p* value	DVD + PN vs. usual care Difference (98.3% CI) *X*^2^ *p* value
	Overall *n* = 371	DVD only *n* = 123	DVD + PN *n* = 120	Usual care *n* = 128				
Either test					**22.81** **<.001**	**19.1%** **(4.4%, 33.8%)** **9.26** **.002** ^a^	9.0% (−4.1%, 22.1%) 2.67 .102^a^	**28.1%** **(14.2%, 42.0%)** **21.55** **<.001** ^a^
No	248 (66.8%)	86 (69.9%)	61 (50.8%)	101 (78.9%)				
Yes	123 (33.2%)	37 (30.1%)	59 (49.2%)	27 (21.1%)				
COL					**22.21** **<.001**	**20.4%** **(7.0%, 33.9%)** **12.6** **<.001** ^a^	3.1% (−8.1%, 14.2%) 0.43 .515^a^	**23.5%** **(10.5%, 36.5%)** **17.67** **<.001** ^a^
No	284 (76.5%)	101 (82.1%)	74 (61.7%)	109 (85.2%)				
Yes	87 (23.5%)	22 (17.9%)	46 (38.3%)	19 (14.8%)				
FIT					2.79 0.248	−1.4% (−11.1%, 8.4%) 0.11 .740^a^	6.0% (3.6%, 14.6%) 2.66 .103^a^	4.6% (−3.9%, 13.1%) 1.68 .195^a^
No	335 (90.3%)	108 (87.8%)	107 (89.2%)	120 (93.8%)				
Yes	36 (9.7%)	15 (12.2%)	13 (10.8%)	8 (6.3%)				

*CI* confidence interval; *COL* colonoscopy; *CRC* colorectal cancer; *DVD* digital video disc; *EMR* electronic medical record; *FIT* fecal immunochemical test; *PN* patient navigation; *X*^2^ chi-square. Significant differences are indicated in bold.

^a^Original *p* value from chi-square test for a screening outcome. *p* value should be compared to the Bonferroni adjusted alpha level of 0.017.

Multivariable modeling supported a strong effect of the DVD + PN; those who received the DVD + PN were more than four times more likely to be screened compared to usual care and 2.5 times more likely to be screened than those who received the DVD alone (see Table 3). Other significant predictors of completion of any CRC screening test included the global mental health and comorbidity scores. Those with higher (better) mental health scores and higher comorbidity scores were more likely to complete any CRC screening test (see Table 3).

**Table 3:** Multivariable Logistic Regression Model of EMR-Documented CRC Screening (Colonoscopy, FIT, or Both)

Variable	Effect^a^	Odds ratio	95% CI^b^	*p* value	Overall *p* value
Study arm	DVD only vs. usual care	1.65	0.80–3.41	.100^c^	**<.001**
	**DVD + PN vs. usual care**	**4.08**	**2.00–8.31**	**<.001** ^c^	
	**DVD + PN vs. DVD only**	**2.47**	**1.26–4.84**	**.001** ^c^	
Age ≥65	Yes vs. No	0.96	0.49−1.87	.906	
Sex	Male vs. female	0.84	0.52−1.37	.490	
Race/Hispanic ethnicity	Non-Hispanic Non-White vs. Non-Hispanic White	1.80	1.05−3.11	.034	.074
	Other vs. Non-Hispanic White	0.91	0.24−3.44	.888	
Years of education		1.03	0.92−1.17	.591	
Having insurance	Yes vs. No	2.21	0.69−7.11	.185	
Global Mental Health scale score		**1.14**	**1.05–1.25**	**.002**	
Comorbidity		**1.08**	**1.02–1.14**	**.007**	

*CI* confidence interval; *CRC* colorectal cancer; *DVD* digital video disc; *EMR* electronic medical record; *FIT* fecal immunochemical test; *PN* patient navigation.

^a^For categorical variables, the reference level is noted after “vs.” For study arm, all three pairwise comparisons are provided.

^b^98.3% confidence intervals are reported for the comparison of intervention arm effects to account for Bonferroni adjustment.

^c^ *p* value for intervention differences should be compared to the Bonferroni adjusted alpha level of 0.017. Significant variables are indicated in bold.

### Quality of Bowel Preparation, Colonoscopy-Related Procedure Anxiety, and Satisfaction With the Procedure

For those participants who completed colonoscopy (*n* = 90) including three participants who completed both colonoscopy and FIT), there were no differences among the three arms in the adequacy of bowel preparation as measured by either the modified Aronchick score (*p* = .665) or the BBPS score (*p* = .503) (see Table 4). Bowel preparation was adequate in 87.8% of all participants with the highest score among the DVD only group (91.7%). Average BBPS scores were reasonably high, ranging from 6.59 to 7.13 out of 9. Colonoscopy-related anxiety and satisfaction with the colonoscopy were assessed only among the 71 participants who completed a colonoscopy and completed these scales. There were no differences among the three arms in colonoscopy-related procedure anxiety (*p* = .559) or satisfaction with the procedure (*p* = .210) (see Table 5).

**Table 4:** Bowel Preparation Scores Between Study Arms for Participants Who Had EMR-Documented Colonoscopy (Including Three Participants Who Had Both FIT and Colonoscopy)

Bowel preparation	Overall *n* = 90	Study arm			*p* value^a^
		DVD only *n* = 24	DVD + PN *n* = 46	Usual care *n* = 20	
Modified Aronchick score: *n* (%)					.665
Inadequate	11 (12.2%)	2 (8.3%)	7 (15.2%)	2 (10.0%)	
Adequate	79 (87.8%)	22 (91.7%)	39 (84.8%)	18 (90.0%)	
BBPS score^b^^,^^c^, mean (*SD*)	6.83 (2.27)	7.13 (1.90)	6.59 (2.40)	7.05 (2.44)	.503

*ANOVA* analysis of variance; *BBPS* Boston Bowel Preparation Scale; *DVD* digital video disc; *EMR* electronic medical record; *FIT* fecal immunochemical test; *PN* patient navigation; *SD* standard deviation.

^a^ *p* values from chi-square test or ANOVA.

^b^ *n* = 1 missing for usual care.

^c^BBPS is a single item ranging from 0 to 9. Higher scores indicate greater quality of bowel preparation.

**Table 5:** Self-reported Colonoscopy-Related Procedure Anxiety and Satisfaction by All Participants Who Had an EMR-Documented Colonoscopy and by Study Arm

Variable	Overall *n* = 71^a^ Mean (*SD*)	DVD only *n* = 16 Mean (*SD*)	DVD + PN *n* = 40 Mean (*SD*)	Usual care *n* = 15 Mean (*SD*)	*p* value^b^
Colonoscopy-related procedure anxiety^c^	2.29 (0.71)	2.46 (0.78)	2.23 (0.67)	2.28 (0.76)	.559
Satisfaction with the colonoscopy experience^d^	3.45 (0.89)	3.38 (0.96)	3.60 (0.78)	3.13 (1.06)	.210

*ANOVA* analysis of variance; *DVD* digital video disc; *EMR* electronic medical record; *PN* patient navigation; *SD* standard deviation.

^a^ *n* = 87 with EMR-documented screening colonoscopy; *n* = 6 missing survey data for DVD only; *n* = 6 missing survey data for DVD + PN; *n* = 4 missing survey data for usual care.

^b^ *p* value from ANOVA.

^c^Colonoscopy-related procedure anxiety: 6 items with possible scores ranging from 1 to 4; higher scores indicate greater anxiety when thinking about having a colonoscopy.

^d^Satisfaction with the procedure: single item ranging from 1 to 4 where 1 = not at all satisfied, 2 = a little satisfied, 3 = mostly satisfied, and 4 = completely satisfied.

## Discussion

Significant disparities in CRC incidence and mortality exist for racial/ethnic minorities, people with less education, and lower incomes leading to higher incidence and mortality rates in these groups [70]. Differences in access to screening and treatment account for more than half of the racial disparity in CRC mortality rates [71–73]. Other contributing factors to disparities for both African Americans and those with low socioeconomic status include unequal access to and receipt of quality treatment and lower rates of participation in CRC screening [74, 75]. Improving CRC screening rates among racial/ethnic minorities has been identified as the first target to reducing these disparities [74, 76].

Participants in this study were unique in that they had been referred by their healthcare provider, and scheduled for, a screening colonoscopy but did not attend their appointment. Some patients called the endoscopy department to cancel their appointment but did not reschedule; however the majority simply did not attend. Findings that participants who received the DVD plus PN intervention were over four times more likely to be screened than the usual care group is consistent with past literature on the positive effect of PN on screening outcomes. PN by telephone provides an easily accessible approach to reaching patients to provide encouragement and support. Although the DVD alone did not significantly increase CRC screening completion overall, the percentage of those who actually viewed the DVD had a higher screening completion rate compared to those who did not view it. The DVD may have provided preliminary information that enhanced the PN effect and supported tailored discussion about an individual’s barriers for either colonoscopy or FIT. Additionally, the mailing of an FIT kit in the DVD + PN group may have enhanced the overall effect of the combined intervention. While the additional power of having patients view a DVD prior to PN is unknown, the fact that the DVD plus PN intervention had the strongest effect suggests that safety net health systems should consider implementing PN with patients who miss their colonoscopy appointments, at a minimum, with or without the DVD.

For participants who completed colonoscopy, neither intervention improved quality of bowel preparation, satisfaction with the colonoscopy procedure, nor reduced colonoscopy-related anxiety. Bowel preparation scores were relatively high across all three arms and similar to those reported in other studies [66, 77, 78] so observing significant improvements may not have been possible. In some settings and with some populations, inadequate bowel preparation is a significant problem requiring patients to repeat the preparation and return on another day. It is also costly for the healthcare system. For many patients, colonoscopy is anxiety provoking. While we anticipated that our interventions would decrease anxiety and increase satisfaction by preparing patients for the test, neither intervention had an effect on those outcomes.

Several studies conducted by our team and others tested similar interventions that effectively increased CRC screening uptake [24, 26, 44, 58, 79–81]. Prior randomized trials of computer-tailored interventions and telephone-based PN conducted by our team showed that these approaches are highly effective at increasing CRC screening [24, 26, 58, 80]. PN has consistently been shown to be effective at increasing completion of screening colonoscopy, especially among minority and low-income populations [40, 44, 48–50, 52, 79]. However, none of these earlier studies tested interventions with patients who have canceled or missed their screening colonoscopy appointments. Rates of colonoscopy completion in the randomized trials cited increased by 10% [40], 41% [48], 28% [49], 13.4% [50], and 11% [52]. Our almost 50% rate of CRC screening completion is higher than most prior studies.

Recent studies have reported promising results of interventions to promote colonoscopy attendance using technology, primarily text messaging or short message services (SMS) [82, 83]. Lam et al. enrolled 2,225 patients scheduled for colonoscopy for any indication and randomized patients to receive a single SMS reminder 7–10 days prior to their appointment that included bowel preparation instructions. The nonattendance rate was significantly lower in the SMS groups compared to controls (8.9% vs. 11.9%, *p* = .02), but the quality of bowel preparation was not significantly different. A timed-release text message navigation program was tested in a randomized trial with 1,625 patients who were scheduled for colonoscopy for any reason, not just screening. Solonowicz et al. tested an intervention consisting of 20 text messages starting 14 days prior to the scheduled colonoscopy. Colonoscopy nonattendance rates were significantly lower in the SMS group compared to controls (8% vs. 14%; *p* < .0001) and rates of adequate bowel preparation for screening colonoscopies were higher in the SMS group (93% vs. 88%, *p* = .04) [83]. SMS-based communication shows promise for increasing attendance at screening colonoscopy appointments.

## Limitations and Future Directions

Participants were recruited from a single safety net health system in the Midwest that served predominantly low-income and minority populations. These findings may not be observed in health systems that serve different populations or that use different approaches to CRC screening. The sample recruited for this study included patients who were scheduled for a screening colonoscopy but had not followed through with the appointment. Therefore, these patients may have been more resistant to CRC screening than the general screening-eligible population, thus making it more difficult to observe an effect. For some participants who remained reluctant to have a screening colonoscopy, offering stool-based testing (less costly, more convenient, noninvasive) was a more acceptable option. It is possible that some patients had actively decided they would not be screened with colonoscopy or with any screening test at this time. The need to continue to counsel/educate patients about the importance of screening as well as assessing and reducing individual barriers to screening is indicated. Some patients may need to have several discussions with their provider before they are ready to complete screening.

At the onset of the study in 2016, this safety net population had very limited access to computers with internet access. We created the intervention using a DVD platform (which requires different programming than a computer or web-based product) because we could easily provide portable DVD players to those who did not have access to a DVD player. Much later in the study, we created a web-based version of the program that is available in both English and Spanish that we made available to the health system after the study was completed. Those versions are currently available at http://mycolonhealth.iupui.edu. The lack of Hispanic participants, a low-income, minority population served in this safety net health system, is another limitation of this study. The Hispanic patient population was relatively small and we did not have the resources to translate all study materials into Spanish or have a Spanish-speaking PN prior to launch. We translated the DVD into Spanish after the study was completed.

We did not have a study arm that received the PN intervention alone, so it is not possible to know what effect receipt of the PN alone would have had. Future studies should examine the impact of PN alone on CRC screening test completion in this population. Understanding the receipt, uptake, and satisfaction with any intervention is essential to interpreting the results [60, 84]. We conducted a process evaluation to assess these factors by interviewing 243 participants randomized to the interventions; only 194 (80%) completed the process evaluation interview and reported viewing the DVD. Forty-nine (20%) participants were unable to be reached by phone to complete the process evaluation interview but were not significantly different from those who did demographically [60]. However, we do not know what proportion of participants who were unable to be reached actually viewed the DVD.

Since the COVID-19 pandemic, changes to CRC screening occurred during that time that may have changed preferences for CRC screening and acceptance of telehealth interventions. Many primary care providers pivoted to promoting home-based screening using stool testing as an alternative to colonoscopy, making this alternative screening method more readily available. Given the equivalence of FIT versus colonoscopy in reducing mortality in average risk individuals and the increased focus on shared decision making, promotion of the patient’s preferred test has potential to increase overall CRC screening. Mailing FIT kits to patients’ homes has proven to be an effective intervention to increase CRC screening and might be used to increase CRC screening with persons who fail to attend a colonoscopy appointment [85–88]. Future studies are needed to reassess patient preferences for CRC screening post-COVID.

## Conclusions

Among minority and low-income patients in a safety net health system who canceled or did not attend a scheduled colonoscopy, the combination of a mailed, tailored DVD plus telephone-based PN was highly effective in moving them to complete CRC screening. Health systems in which nonattendance at scheduled colonoscopies is a problem should consider implementing telephone-based PN, with or without small media, to increase completion of CRC screening.
